# Oligo-Metastatic Cancers: Putative Biomarkers, Emerging Challenges and New Perspectives

**DOI:** 10.3390/cancers15061827

**Published:** 2023-03-17

**Authors:** Alessandro Ottaiano, Mariachiara Santorsola, Luisa Circelli, Anna Maria Trotta, Francesco Izzo, Francesco Perri, Marco Cascella, Francesco Sabbatino, Vincenza Granata, Marco Correra, Luca Tarotto, Salvatore Stilo, Francesco Fiore, Nicola Martucci, Antonello La Rocca, Carmine Picone, Paolo Muto, Valentina Borzillo, Andrea Belli, Renato Patrone, Edoardo Mercadante, Fabiana Tatangelo, Gerardo Ferrara, Annabella Di Mauro, Giosué Scognamiglio, Massimiliano Berretta, Maurizio Capuozzo, Angela Lombardi, Jérôme Galon, Oreste Gualillo, Ugo Pace, Paolo Delrio, Giovanni Savarese, Stefania Scala, Guglielmo Nasti, Michele Caraglia

**Affiliations:** 1 Istituto Nazionale Tumori di Napoli, IRCCS “G. Pascale”, Via Mariano Semmola, 80131 Naples, Italy; 2 AMES, Centro Polidiagnostico Strumentale SRL, Via Padre Carmine Fico 24, 80013 Casalnuovo Di Napoli, Italy; 3 Oncology Unit, Department of Medicine, Surgery and Dentistry, University of Salerno, 84081 Baronissi, Italy; 4 Department of Clinical and Experimental Medicine, University of Messina, Via Consolare Valeria, 98125 Messina, Italy; 5 Coordinamento Farmaceutico, ASL-Naples-3, 80056 Ercolano, Italy; 6 Department of Precision Medicine, University of Campania “L. Vanvitelli”, Via Luigi De Crecchio 7, 80138 Naples, Italy; 7 INSERM, Laboratory of Integrative Cancer Immunology, 75006 Paris, France; 8 Equipe Labellisée Ligue Contre le Cancer, 75006 Paris, France; 9 Centre de Recherche des Cordeliers, Sorbonne Université, Université de Paris, 75006 Paris, France; 10 SERGAS (Servizo Galego de Saude) and NEIRID Lab (Neuroendocrine Interactions in Rheumatology and Inflammatory Diseases), Research Laboratory 9, IDIS (Instituto de Investigación Sanitaria de Santiago), Santiago University Clinical Hospital, 15706 Santiago de Compostela, Spain

**Keywords:** oligo-metastatic, metastases, genetics, prognosis, radiotherapy, biomarkers

## Abstract

**Simple Summary:**

Oligometastatic disease is a condition in oncology where cancer affects only a few distant sites. It is associated with a low-burden spread and a more favorable prognosis compared to polymetastatic disease. Recent studies have identified specific molecular and genetic features that underlie the oligometastatic phenotype, including reduced cancer cell migration and invasion ability, and an enhanced immune response in the metastatic microenvironment. Understanding these characteristics could suggest innovative personalized therapies and contribute to improving the understanding of complex cancer–host relationships. This scoping review highlights new clinical, biological, and methodological challenges that characterize this fascinating field from a modern and innovative perspective. By shedding light on the unique features of oligometastatic disease, we aim to promote the development of more effective and tailored treatments for patients with this condition.

**Abstract:**

Some cancer patients display a less aggressive form of metastatic disease, characterized by a low tumor burden and involving a smaller number of sites, which is referred to as “oligometastatic disease” (OMD). This review discusses new biomarkers, as well as methodological challenges and perspectives characterizing OMD. Recent studies have revealed that specific microRNA profiles, chromosome patterns, driver gene mutations (*ERBB2*, *PBRM1*, *SETD2*, *KRAS*, *PIK3CA*, *SMAD4*), polymorphisms (*TCF7L2*), and levels of immune cell infiltration into metastases, depending on the tumor type, are associated with an oligometastatic behavior. This suggests that OMD could be a distinct disease with specific biological and molecular characteristics. Therefore, the heterogeneity of initial tumor burden and inclusion of OMD patients in clinical trials pose a crucial methodological question that requires responses in the near future. Additionally, a solid understanding of the molecular and biological features of OMD will be necessary to support and complete the clinical staging systems, enabling a better distinction of metastatic behavior and tailored treatments.

## 1. Introduction

The metastatic spread to distant organs such as lungs, liver, bone, and brain from primary tumor is the leading cause of death in cancer patients. The number, size of tumor lesions, and involvement of loco-regional lymph nodes are well-established methods of measuring the level of cancer diffusion, and they drive clinical staging systems for cancer [1,2,3]. However, the initial tumor burden may vary in clinical practice in the context of metastatic disease, typically stage IV disease [4]. Some patients present at diagnosis with a highly metastatic pattern involving multiple sites (“poly-metastatic disease”), while others have a more indolent course of the disease with involvement of a lower number of sites (“oligo-metastatic disease” or OMD) [5]. In recent years, the study of OMD has gained increasing attention since it is becoming clear that a multidisciplinary treatment approach and long-term therapeutic path can achieve extended disease control in OMD patients. This review will present and discuss the most recent insights, challenges, and perspectives about OMD, ranging from practical definitions and clinical contexts to new potential biomarkers.

## 2. The Origin of OMD: The Concept of “Metastatic Virulence”

It is heavily debated that OMD is not merely a temporary or intermediate status between a localized and low-burden disease and a diffuse one, but rather a distinct disease with specific biological and molecular features. In other words, the low “metastatic virulence” of OMD may reflect specific and dynamic states of tumor biology and/or host/tumor relationships [6]. The complex and multi-step process that accounts for the acquisition of a full malignant phenotype (from primary tumor to increasingly aggressive and poly-metastatic) can be divided into a large number of biological features, such as epi/genomic instability, epithelial-to-mesenchymal transition (EMT), proliferation, self-renewal, invasiveness, interrelation with lymphocytes and tumor environment, life in transit, organ-specific homing, neo-angiogenesis, and so on [7,8,9,10,11,12]. However, the acquisition of full properties for each of these phenomena cannot be dichotomously identified (genomic instability: yes/no; EMT: yes/no; proliferation: yes/no; etc.), as they plausibly work continuously. Oligo-metastatic cells may have a lower capability in one or more of these necessary characteristics compared to the poly-metastatic cells. Therefore, the origin of oligo-metastases could rely on reduced metastatic power (i.e., “metastatic virulence” scarcely and elusively valuable). An attractive consequence of these considerations is that clinical staging systems should differentiate oligo-metastatic cancer forms and ideally include specific molecular characteristics [13]. 

## 3. OMD Clinical Contexts

Previous studies have provided a pragmatic and quantitative approach to defining OMD. Specifically, oligo-metastases can refer to 1–3 metastatic tumors per organ with a maximum size of less than 7 cm [14]. A recent study provided a more stringent definition with a maximum of 1–5 tumors and a size limit of 5 cm [15]. This definition should also take into account the “rate of metastatic growth”, which is slower in OMD [16], but it is difficult to quantify and poorly applicable in clinical practice. On the other hand, many authors suggest that OMD could be defined as the presence of a metastatic cancer amenable to curative/radical therapeutic local interventions (surgery or radiotherapy) on all metastatic lesions, regardless of their number and/or volume [5,17]. However, in the real world, identifying OMD is often done retrospectively, as many patients who undergo radical treatment of oligo-metastases develop aggressive, poly-metastatic diseases within a year, while others never experience disease progression (true OMD).

A recent consensus study by ASTRO/ESTRO (American Society for Radiation Oncology/European Society for Radiotherapy and Oncology) [18] has provided clear and practical definitions for the various clinical scenarios of OMD. It is crucial to know and understand these definitions not only for the sake of scientific terminology, but also because they will require prospective validation and prompt scientific exploration in the near future.

The OMD classification and nomenclature consists of:

- Genuine (or “de novo”) OMD: it is considered the “purest” phenotype of OMD, when the cancer has no prior history of polymetastatic disease. It is useful to distinguish between synchronous and metachronous OMD, which refer to the diagnosis being made within or after 6 months of the primary cancer diagnosis, respectively.
- Induced OMD: the polymetastatic cancer has become limited to a small number of metastatic sites (OMD) following systemic treatment.
- Repeat OMD: OMD that recurs after a previous diagnosis and treatment for OMD.
- Repeat and induced can be associated with different imaging dynamics (i.e., repeat oligo-recurrence vs. induced oligo-recurrence; both indicate new oligometastatic lesions from OMD or polymetastatic disease, respectively):
- Oligorecurrence: OMD that recurs after initial treatment during a treatment-free period.
- Oligoprogression: the OMD progresses during active systemic treatment.
- Oligopersistence: the OMD persists after initial treatment.

## 4. Epidemiology of OMD

**Figure 1:**
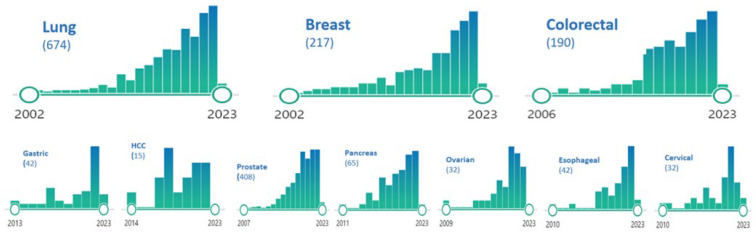
Literature analysis (number per year) of articles dealing with OMD (until January 2023).

The incidence of OMD varies depending on the type of tumors. It was hypothesized in the past that some cancers such as pancreatic adenocarcinoma and small-cell lung cancer, never present with an oligo-metastatic behavior; to date, an oligo-metastatic status has been reported in all cancers [19,20,21,22,23,24,25,26,27,28,29,30,31,32]. The incidence of OMD varies greatly among cancer types, ranging from extremely rape (such as SCLC where only a few case reports are described) [21] to 10–40% of HCC [25,26]. Table 1 presents the incidence of OMD for each cancer type. A literature analysis was performed to provide an overview of the research on OMD, which is represented in Figure 1. The search was conducted in PubMed (accessed on 9 February 2023) using the keyword “oligometastatic disease” and related keywords specific to each disease. Although this research is far from systematic (it did not include additional “gray” keywords, multiple databases, or crucial “scoping” studies), the patterns revealed in Figure 1 indicate a trend toward increasing attention to OMD over time, which reaches a peak in the last year. Furthermore, it is important to note that the incidence of “induced” OMD is increasing in all cancers as systemic treatments (biologic drugs, immunotherapies, and integrated approaches) improve. This is a new clinical scenario resulting from the effect of improved initial treatments for primary poly-metastatic cancers.

## 5. Definitive Local Therapies in OMD

The concept of OMD is an intriguing and versatile clinical model that allows for a personalized and multidisciplinary approach to treatment [33,34]. Independently from the origin of the primary tumor, the therapeutic approach to OMD generally tries to make the patient disease-free through surgery and/or less invasive loco-regional techniques, such as radiation therapy.

Although surgical techniques are beyond the scope of this article, it is well established that surgery can be curative in certain clinical situations, such as pulmonary metastases from soft tissue sarcomas, osteosarcomas, and renal cell cancers, or hepatic metastases from colorectal cancer, even when multiple metastases are present. In true OMD cases, repeated surgeries and DLTs (definitive local therapies) have shown a similar likelihood of cure as the initial surgery [35,36,37,38]. Pulmonary and liver metastasectomies are the main surgical treatments for OMD, with notable differences in terms of technical, anatomical, and clinical-prognostic issues. The best prognostic results are achieved when only one site is affected; however, the availability of mini-invasive surgical techniques, including robot-assisted and laparoscopic approaches, has expanded the fraction of patients who may benefit from oligo-metastatic lesion removal, including older or sicker patients with comorbidities [39,40,41,42,43]. However, due to the complexity of some clinical scenarios, such as brain, lymph node, or limited peritoneal involvement, a personalized and multidisciplinary assessment and discussion is required to determine the most appropriate surgical approach.

Stereotactic radiotherapy (SRT) is a highly precise form of radiation therapy used in oncology to treat tumors in specific areas of the body. It delivers high doses of radiation to a precisely targeted area while minimizing exposure to surrounding healthy tissues, achieved through several different techniques. SRT on small, well-defined masses is safe and feasible in areas where other forms of treatment (such as surgery) may be difficult or impossible [44,45]. However, the risk of toxicity may depend on the size and location of the tumor. It is clear that in OMD patients the role of radiotherapy is not a palliative treatment but a disease-modifying treatment. Furthermore, a strong rationale exists to support the addition of SRT in the immunotherapy era. Several data demonstrated that hypo-fractionated SRT is able to increase MHC (Major Histocompatibility Complex) class I expression, to improve APCs (Antigen-Presenting Cells) entry into tumor masses, to activate CTLs (Cytotoxic T Lymphocytes) through increased intracellular peptide generation and cytokine secretion (Interleukin-2 and Interferon-gamma) [46,47,48,49,50,51,52]. These phenomena are on the basis of a “radiation-induced immunity”. However, the role of SRT in OMD continues to evolve. Future directions for the treatment of OMD include the development of new SRT techniques for improved targeting and the use of SRT in combination with other forms of less intensive and biologic cancer treatment, such as immunotherapy. In fact, the time for the use of biological or target-oriented drugs as monotherapy in OMD is not yet ripe as the biology of this type of disease is still largely unknown. 

Alternative treatments to SRT include radio frequency [53,54], electroporation [38], laser interstitial thermal therapy [55], and others [56,57]. The choice of DLT depends on the patient’s medical condition, tumor location, center expertise, and multidisciplinary consensus. These DLTs can achieve comparable control rates and time-to-outcome as surgery, at least in selected populations. 

The main clinical characteristic of oligometastatic patients is that, even with locoregional treatments, their median survival is more than double of polymetastatic patients. In this context, two main clinical courses can be listed. In some cases, OMD progresses slowly, affecting the function of major organs (oligo-progression). However, locoregional treatments can be intermittently and reasonably prolonged until advanced stages of the disease in these patients [58,59]. In other patients, a clear and clinically aggressive polymetastatic disease develops (“poly-metastatic progression”), requiring a classical approach based primarily on the administration of multiple lines of non-cross-resistant systemic chemotherapy. Interestingly, some studies identify the median time to polymetastatic conversion (tPMC) as a measurable outcome associated with treatment efficacy [60].

The practical and most important question on a clinical point of view is: could DLTs be considered a standard therapeutic option in OMD? 

Unfortunately, while DLTs, particularly SRT, are widely used in clinical practice to manage OMD, there is limited evidence to support their use. A review of scientific literature from PubMed in the past year (when a peak of publications is registered) reveals a high number of heterogeneous, retrospective, and small-scale clinical studies conducted in real-world settings [61,62,63,64,65,66,67,68,69]. While these studies are important, their results cannot formally change the standard of therapy. The evidence ranges from case reports and retrospective series [61,62,63,64,65,66,67,68] to phase III randomized trials [69] such as the SINDAS trial. This trial showed that adding SRT to TKIs significantly improves survival in 133 EGFR-mutated NSCLC OMD patients (median OS TKI plus SRT: 25.5 months vs. 17.4 months in TKI monotherapy). The studies range in size from 39 NSCLC OMD patients (in a study reporting an interesting retrospective comparison between DLTs, predominantly radiotherapy, vs. TKIs) [64] to 284 OMD patients with different cancers (a single-arm study performed predominantly in lung, colon, and breast OMD) [67]. The median survivals achieved in these studies with SRT are higher than those reported in the poly-metastatic setting, 30.8 and 53.4 months, respectively, confirming that OMD has a good prognosis. However, examples of systemic approaches have also been pursued and published in the past year in OMD, including immuno-plus chemotherapy [61,62] and immune therapy plus SRT [66]. These studies are retrospective, heterogeneous (patients received different treatments before and/or after the therapeutic approach for OMD), and lacking in comparison with a “control” arm. Only a few prospective randomized trials (Table 2) on the role of SRT (vs observation or standard of care) in OMD are available [69,70,71,72,73], but their results all point in the same direction. Time-to-outcome curves are clearly separated even in longer time follow-up, indicating a persistent and robust beneficial effect of SRT in OMD. However, the optimal dose and schedule for SRT in various OMD situations is still unknown due to the variety in treatment dosage and timing among clinical trials. 

## 6. Biomarkers of OMD

**Figure 2:**
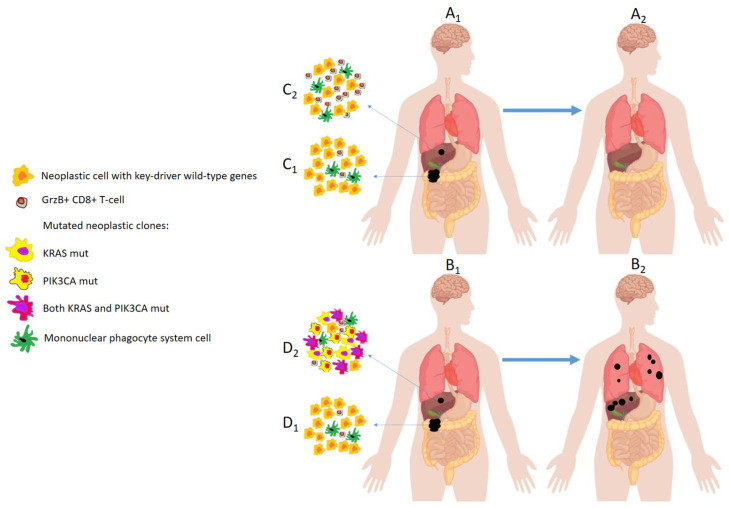
The figure shows two patients (**A_1_**,**B_1_**) who have the same onset of de novo OMD. Both patients receive radical excision of all lesions (primary tumor and single liver metastases). Patient A_1_ is disease-free at the 3-year follow-up (**A_2_**) (“true” OMD). Patient B_1_ develops poly-metastatic disease (“false” OMD) within 1 year of follow-up (**B_2_**). The markers that clearly differentiate these two clinical entities that apparently have the same onset are unknown. Previous evidence suggests that the dynamic study of the primary tumor and metastases (**C_1_** vs. **C_2_** and **D_1_** vs. **D_2_**) could provide important prognostic indications. High infiltrates of cytotoxic granzyme-b positive (GrzB+) CD8+ T cells and the regression of key-driver mutation clones could be the basis for true OMD. The cellular composition of the tumor mass is shown in the figure (**C_1_**,**C_2_**_,_**D_1_**,**D_2_**) to focus on these two last concepts. Some types of cells that make up the tumor microenvironment, such as neutrophils, mast cells, fibroblasts, regulatory cells, etc., have been omitted.

Unfortunately, very few studies have been so far performed to find specific genetic and biologic characteristics of the OMD (Table 3). In fact, it has been considered that investigating the relationship between genotype and phenotype in cancer is extremely challenging. Cancer usually develops as a multi-gene acquired disease with the exception of few uncommon, inherited forms of tumors, such as retinoblastoma and Wilms’ tumor [74,75,76]. The foremost obstacle in performing genotype/phenotype correlations is the selection of appropriate human cancer models. This is because several genes involved in widespread conditions like hypertension, diabetes, allergies, and chronic inflammation contribute to the heterogeneity of cancer [77,78,79,80,81,82,83,84,85]. The latter diseases can interfere with cancer genetics. In fact, some genes involved in cancer-related processes, such as proliferation and angiogenesis, are altered in hypertension and atherosclerotic plaque or are induced due to hypoxia, oxidative stress, and inflammation [86,87]. Lussier et al. reported that specific microRNA profiles from OMD patients drive an oligo-metastatic behavior both in vitro and in vivo [88]. Interestingly, oligomiRNAs target genes are involved in adhesion, invasion, and migration. Although the study was performed in tumor cells from different histologies (colon, small cell lung cancer, non-small cell lung cancer, renal, sarcoma and ovarian cancer), most of oligomiRNAs mapped at a common locus (14q32), suggesting that common epigenetic/genetic phenomena are responsible for OMD. In a large study on clear cell renal carcinoma (575 primary tumors and 335 matched metastases), somatic copy number alterations, genetic intra-tumor heterogeneity, chromosome 9p status associated with the oligo-metastatic and good prognosis behavior of clear cell renal carcinoma [89]. Furthermore, as already reported in other settings, in contrast with the common knowledge suggesting that gene mutations constantly prompt cell transformation and metastases, some driver gene mutations (*PBRM1* and *SETD2*) were associated with attenuated progression and OMD. More studies have been reported in colorectal cancer (CRC) where molecular subtyping (“canonical” and “immune” subtypes) [90], regression of key-driver gene mutations (*KRAS*, *PIK3CA*) [91], high level of T-cell infiltration into metastases [92], high level of peripheral cytotoxic T-cells [93], specific gene mutations (*ERBB2*) [92] correlates with the OMD phenotype. In particular, in our previous reports [91,92], we provided an evolutionary and dynamic analytic perspective, highlighting that comparing primary and metastatic lesions could assist in identifying the true de novo oligo-metastatic behavior. Specifically, we found that patients with a “regressive” genetic trajectory from primary to metastatic lesions and high granzyme-B, CD8+ T cell infiltration into the tumor core of metastatic lesions did not experience relapse within 3 years of follow-up. In contrast, patients who did not exhibit these characteristics developed poly-metastatic disease within 1 year of radical resection of all visible lesions (Figure 2). 

Interestingly, our previous works [91,92] on oligo-metastatic CRC patients focused on identifying patients who only had cancer as their illness and characterizing the genetics of all their lesions. Previous studies suffered from extreme heterogeneity, including different stages, treatments, comorbidities, and more, which can impact the interpretation of results. Specifically, we studied patients who only had lung- or liver-limited single metastatic nodules. To identify the most dominant and interrelated genes in these patients, we used the Phenolyzer tool [94]. Interestingly, we found that in addition to APC and TP53, EP300 was among the top three dominant genes. *EP300* encodes a histone acetyl-transferase involved in regulating chromatin activity and can influence important cell processes like proliferation and differentiation [95]. Although *EP300* mutations have been found in many cancers, including CRC, its role in tumorigenesis is debated and contradictory. Our results suggest that further research is needed to define the relationship between *EP300* and oligo-metastatic behavior. In addition to genes involved in proliferation, apoptosis, differentiation, and neoangiogenesis, a significant number of other genes were identified in the group with “true” de novo OMD. These genes were involved in DNA repair mechanisms, including *MSH3*, *BRCA1*, *ATM*, *POLE*, *BRCA2*, *CHEK1*, and *GLI1*. Increased MSI and TMB, along with these genes, may account for the high immunogenicity of metastases in this group of patients who never developed poly-metastatic disease in subsequent follow-up. On the other hand, patients who developed poly-metastatic disease after radical resection of all their lesions showed a marked mutational divergence, with only one shared gene: *RP11-145E5.5*. This gene encodes a S-methyl-5′-thioadenosine phosphorylase (MTAP) involved in polyamine biosynthesis [96]. Although loss of MTAP activity has been hypothesized to play a role in malignant melanoma, little is known in CRC, where it appears overexpressed compared to normal mucosa and positively related to aggressiveness of CRC cells [97]. We also observed frequent alterations of genes correlated to the homing of metastases to the liver, including *HSP90AA1*, *NR4A2*, *KDR*, *FLT3*, and *RPS6KB2*. These genes can act directly by promoting cell migration, EMT promotion, and proliferation, or indirectly through pleiotropic actions like protein stabilization, epigenetic modifications, and protein synthesis.

Genetic changes in lung-limited oligo-metastatic patients were identified in *EpCAM* (Epithelial cell adhesion molecule), *TP53*, *caspase-8*, and *ERBB2*, which are considered to be significant and frequently shared among the patients. Notably, both *EpCAM* and *caspase-8* play a role in regulating cell proliferation, migration, and adhesion to lung tissue [98,99]. Hence, their alteration could be responsible, at least in part, for the lung homing of metastatic cancer cells. *ERBB2* was also found to be frequently mutated, with a non-synonymous coding variant, p.Pro1170Ala, which may alter the spatial conformation of the tail region and affect tyrosine kinase activity [100]. *ERBB2* is a member of the *ERBB* family of membrane tyrosine kinase receptors, which includes *EGFR*, *ERBB3* (kinase domain-lacking), and *ERBB4*. While no ligands for ERBB2 have been identified, it can hetero-dimerize with any of the other three ERBB family receptors upon ligand binding. This hetero-dimerization activates autophosphorylation of cytoplasmic tyrosine residues, which then bind various signaling molecules involved in proliferation, migration, and angiogenesis [101]. *ERBB2* amplification has been extensively studied in cancer, but very little is known about the role of point mutations. Interestingly, in *ERBB2* overexpressing breast cancer cell lines, lung colonization is predominant and mediated by SPARC (secreted protein acidic and rich in cysteine) [102,103].

**Table 3:** Biomarkers identifying the oligo-metastatic status.

Author, Year	Tumor Type	Biomarker	Clinical Significance
Lussier, 2011 [88]	Mixed tumor histologies.	OligomiRNAs.	MicroRNAs expression patterns associated with OMD.
Turajlic, 2018 [89]	Clear-cell renal cell carcinoma.	9p loss. Low intra-tumor heterogeneity of primary cancer. High genomic somatic copy-number alterations.	The patients with these characteristics develop poly-metastatic disease.
		*PBRM1* and *SETD2* mutations in primary tumor.	These genetic features associate with oligo-metastases and attenuated progression.
Pitroda, 2018 [90]	Colorectal cancer.	“Canonical” and “immune” molecular subtypes in primary tumor.	They associate with long-term survival and OMD.
Ottaiano, 2020 [91]	Colorectal cancer.	*KRAS* regression from primary to metastatic lesions. *ERBB2* p.Pro1170Ala.	They associate with lung-limited OMD.
Ottaiano, 2020 [92]	Colorectal cancer.	Loss of *KRAS* and *SMAD4* alterations from primary to metastatic lesions. High granzyme-B+ T-cell infiltration into metastatic tumor.	The patients with these characteristics remain with liver-limited OMD for long time.
		Gain in *KRAS*, *PIK3CA* and *SMAD4* alterations. Scarce granzyme-B+ T-cells infiltration.	The patients with these characteristics develop poly-metastatic widely diffusive disease.
Ottaiano, 2022 [93]	Colorectal cancer.	*KRAS* regression from primary to metastatic lesions. HLA-C7 aplotype.	The patients with these characteristics remain oligometastatic for long time.
Ottaiano, 2022 [103]	Colorectal cancer.	Absence of *TCF7L2* variants, low frequency of type 2 diabetes-associated genetic polymorphisms.	The patients with this characteristic have persistent OMD.

Moreover, we previously reported that certain genes associated with type 2 diabetes (T2D) may also play a role in the malignant phenotype of OMD in CRC [104]. In particular, some variants associated with T2D, such as *HNF1A* p.I27L, *IDE3* p.T105A, *IRS1* p.S892G, and *INSR* p.A2G, although considered benign, could influence the activity of related proteins. This effect can also be found in changes at 5′-UTR or intron variants that influence transcription activity or alternative splicing. The genetic results of the OMD setting of CRC indicate that these genetic variants (polymorphisms) were less prevalent compared to the poly-metastatic disease. This observation adds further complexity to the phenotype of cancer transformation processes and OMD phenotype. In fact, the effects of these variants are unknown and largely undervalued from both a functional and clinical perspective. Interestingly, diabetes-associated *TCF7L2* variants were absent in the observed group of patients with OMD from CRC. TCF7L2 is a transcription factor that plays a role in various pathways involved in CRC and acts as an effector in the Wnt pathway [105]. The *TCF7L2* gene is strongly associated with T2D and is located on chromosome 10q25.3, with rs7903146 being one of the most common single nucleotide polymorphisms in the *TCF7L2* gene. The exploration and identification of molecular characteristics of OMD is a crucial endpoint. Clinical staging systems are imperfect and too simplified models. 

In the future, a solid understanding of molecular and biological features will necessarily support and complete the clinical information to distinguish the metastatic range. Although defined only by the tumor burden, the OMD represents a setting where the discovery of biomarkers will dramatically change the clinical management and enter into clinical staging systems. 

## 7. Timings of DLTs and Systemic Treatments

The timing of surgery and other forms of DLTs is a highly debated topic. Patients who develop new oligometastases within a year after surgery should be not recommended for repeated surgery but considered for other forms of DLTs [106]. However, there is a lack of randomized trials that could establish any survival differences or clarify the role of pseudo-adjuvant systemic interventions after surgery and/or other DLTs in OMD. To address this gap, randomized trials are essential to explore the role of adjuvant interventions at least in de novo OMD patients treated with DLTs. On a practical point of view, clinicians consider multiple factors driving the decision about chemotherapy and its type, such as the previous intensity and lasting of response, time-to-progression, associated symptoms, tolerability of the current and next line of systemic treatments, predicted efficacy of the next line of systemic treatment, patients’ needs and expectations, age, and comorbidities. Even if the scientific community perceives OMD as being amenable to “less intensive” treatments, no randomized studies have been published on such approaches. A debated issue in this regard is deciding the best control arm, which can be observation or standard systemic treatment. However, in the absence of biomarkers specifically and unequivocally identifying OMD, this induces difficult methodological, ethical, and pragmatic problems.

## 8. Technological Limits for Studying OMD

Exploration of genetic characteristics is essential to understand the biological dynamics of OMD. The discussion about the limitations of current approaches can help to interpret the existing knowledge and to overcome these limitations. Next Generation Sequencing (NGS) technology has generated great enthusiasm in the scientific community due to its ability to sequence DNA from restricted gene panels to entire genomes quickly and at a relative low cost [107,108]. This breakthrough was made possible by integrating biochemistry, molecular technology, and bioinformatics. The potential impact of NGS in cancer research is remarkable since tumor is a complex multi-gene disease. Therefore, the identification of genes involved in malignant transformation and progression is critical to design effective treatments. In most clinical and experimental settings, researchers have to analyze a cancerous tissue, either fresh or paraffin-embedded, selected by the pathologist. The pathologist enhances the analyzed tissue by extracting the most cancer-enriched areas through macro and micro dissection. The minimum tumor cell content for adequate NGS analysis is typically 20%, particularly when the aim is to find specific mutations [109]. When exploring the “genetic landscape” of a tumor, efforts should be made to minimize contamination with normal cells to avoid interference and accurately estimate the variant allele frequency (VAF) of tumor genetic variants. In this scenario, obtaining more than 95% neoplastic cells should be ideal. Two closely related mechanisms can affect the study of OMD genetics: heterogeneity and evolution. When a tumor develops, it adapts itself to the host through genetic evolution, and most malignant cancers progressively acquire and accumulate alterations in genes related to DNA integrity and stability [110]. These genetic changes increase cancer mutational plasticity and heterogeneity, making tumor cells dynamic evolutionary machines [111]. Heterogeneity and genetic dynamism are present and crucial in most malignant tumors and are responsible for the clinical/phenotypic trajectories of cancer, such as OMD. Firstly, neoplastic “OMD cells” can be “quantitatively” scarce in the primary tumor and result in a low VAF in genetic NGS assessments, inducing a high risk of analytic biases and underestimation. It is crucial to analyze these cells as they contain genetic information that is likely linked to the OMD phenotype. Moreover, analyzing only the primary tumor, due to healthcare budget limitations or unavailability/inaccessibility of metastatic tissue, can cause the loss of detection of these genetic alterations wrongly classifying them as “metastatic private events” with low significance and penetration. These crucial events, which occur during the early stages of the malignant process, are elusive and diluted by other genetic alterations and polymorphisms. A single NGS assessment is a single genetic snapshot.

More dynamic and complete (primary and metastatic lesions) assessment of the tumor is required to allow large-scale genetic exploration of OMD. To this regard, improvements in sequencing platforms, technologies, and bioinformatics are required. We are witnessing the development of less invasive, new digital high-throughput PCR platforms based on the combination of PCR techniques and cytofluorometric assays and nanotechnology-based biosensors capable of detecting mutated circulating tumor DNA directly at the patient’s bedside [112,113]. These advancements will allow for precise (single cell) and dynamic (repeatable) determination of OMD. Furthermore, these techniques can be applied to patient tissues, blood, or even biological fluids such as saliva and tears. The so-called liquid biopsy can disclose a new scenario in which the sum of all the gene mutations in the primary and metastatic tumor can be detected at the same time giving a general picture about the mutational landscape of the neoplasm. Moreover, it can be repeated during the course of the disease and after the treatments that can, in turn, induce a mutational pressure to the tumor changing the genetic and biological behavior. The technological advancements in NGS today give the opportunity to analyze the circulating tumor DNA at high sensitivity and specificity.

## 9. Exploring the OMD from Cancer Biopsies: Pros and Cons

The analysis of DNA from cancer biopsies is a promising approach for understanding the molecular basis of OMD and developing personalized treatment strategies. However, there are several challenges associated with this approach. Tumor heterogeneity is one such challenge, as tumors comprise a diverse range of cell types, each with a unique genetic landscape [114]. Biopsies can provide a limited view of the tumor’s genetic profile, where important mutations may be missed. To overcome this challenge, multiple biopsies from different areas of the tumor can be taken, or advanced techniques such as single-cell sequencing can be employed for a more comprehensive view of the tumor’s genetic landscape [115]. Another factor that can affect the quality of DNA analysis is the type of tissue sample. Fresh tissues are preferred as they contain high-quality DNA and RNA that can be used for downstream applications [116]. However, fresh samples may be more difficult to obtain and manage logistically, and paraffin-embedded tissues may be the only option available in some cases [117]. These samples are often degraded, which can pose technical challenges during DNA analysis, such as low DNA yield, poor quality DNA, and increased levels of artifacts. Therefore, careful consideration of the tissue type and appropriate techniques for DNA analysis is crucial to obtaining reliable and accurate results. Furthermore, the dynamic nature of cancer adds another layer of complexity to the analysis of DNA from cancer biopsies in OMD. A single biopsy from a single lesion may not be representative of the entire tumor’s genetic profile, as tumors are constantly evolving and changing over time [118]. This is particularly important in the context of the differentiation between oligo- and poli-metastatic disease, where a single biopsy may not accurately reflect the genetic landscape of all metastatic sites. Serial biopsies taken over time can track tumor evolution and identify potential therapeutic targets. Analyzing DNA from cancer biopsies has both advantages and challenges, with tumor heterogeneity, tissue type, and cancer dynamics being critical factors to consider. 

## 10. Does Chemotherapy Induces Genetic Remodeling in OMD?

OMD is a stage of cancer in which there is limited spread of cancer cells to distant organs. OMD presents with low burden metastatic involvement, making it possible to apply therapeutic strategies based on pre-operative and/or peri-operative chemotherapies. This is particularly relevant on a clinical point of view in liver metastases from colorectal cancer [119]. Surgical resection is the primary treatment for oligo-metastatic CRC, especially in the initially resectable liver metastases. However, the role of chemotherapy in this setting is becoming increasingly important. The folfox schedule, a chemotherapy regimen that combines fluorouracil, leucovorin, and oxaliplatin, has been shown to be effective in treating liver metastases from colorectal cancer. Nordlinger et al. demonstrated that the use of folfox in the neoadjuvant setting resulted in a higher rate of complete resection and longer disease-free survival compared to surgery alone [120]. This suggests that the use of chemotherapy in OMD can improve outcomes and increase the chances of complete and durable remissions. However, reflection and insights are necessary since the study of the molecular and genetic effects of chemotherapy in OMD is an important area of research. Two observations need to be made: this study did not demonstrate improvements in survival [121], and the use of chemotherapy can induce genetic changes in cancer cells. Understanding these changes can lead to the development of new therapies that target specific genetic mutations and help identify patients who are most likely to respond to treatment. For example, the response to chemotherapy can be influenced by mutations in genes that regulate DNA repair, such as BRCA1 and BRCA2. Patients with mutations in these genes may be more sensitive to chemotherapy that induces DNA damage [122]. 

In this regard, our previous works revealed an important and intriguing observation [91,92]. Specifically, we found a significantly higher occurrence of private events in metastatic lesions (with a genetic concordance <20% in all coding variants) in patients who underwent chemotherapy (fluoropyrimidines and oxaliplatin) prior to surgical resection of metachronous lung metastases. These findings suggest a compelling hypothesis that the treatment may contribute to the genetic heterogeneity of subsequent cancer cells that progress. Further research is necessary to clarify and explore these phenomena and how they can be exploited for personalized therapies.

## 11. Heterogeneity of Initial Tumor Burden in Clinical Trials

It is clear that patients with OMD give an intriguing opportunity to study low-metastatic potential/low-burden metastatic cancer. OMD has a favorable prognosis as the disease is controlled effectively with local treatments and milder systemic therapies. In fact, the median survival of low-burden CRC patients is typically higher (around 44 months) compared to that of patients with poly-metastatic disease (24 months) [123]. A crucial scientific question is how the initial burden of metastatic disease, including the presence of OMD, is reported in phase III clinical trials. We previously performed a systematic review of phase III randomized clinical trials in NSCLC, breast, and colorectal cancers to assess the reporting and analysis of the initial tumor volume of enrolled patients, including any OMD [4]. Interestingly, we found no clear identification of OMD in the analyzed trials. In 28.6% of the trials, a “low-burden disease” was reported, but this was mostly based on the number of affected organs without any further information on the extent of the disease. This cannot accurately define a patient having OMD. In fact, a patient with only one affected organ could have a larger number and size of metastatic lesions than another with multiple involved sites. Moreover, only a limited number of trials used the extent of the disease as stratification factor, and no trials used an explicit definition of OMD as an exclusion criterion. In some trials, particularly in lung cancer, stage III vs. IV was used as a stratification factor, but a patient with OMD (stage IV) may have a smaller tumor burden than another with extensive stage III loco-regional lymph nodal disease. In 18.6% of the trials, subgroup analyses did not consider the oligo- vs. polymetastatic status, but only the high vs. low disease burden, which was heterogeneously defined. Most importantly, if the size of low-burden disease (or OMD) patients in different study arms is unequal, this could introduce unexpected biases into the results. In 2 studies, we demonstrated a significant imbalance between arms in patients with low-burden disease, increasing the probability of biased results. 

Could the unbalanced enrollment of genuine OMD patients affect the results of trials and the prognosis of patients? This is a crucial methodological question that remains unanswered. As the analyzed trials did not address this issue, the direct impact of an imbalanced distribution of OMD patients between treatment arms cannot be measured. To shed light on this question, we collected information on the treatment and outcomes of 112 consecutive mCRC patients with characteristics allowing inclusion in phase III trials. We found a statistically significant difference in survival between polymetastatic and OMD patients. Some patients were indeed enrolled in clinical trials, and the presence of omCRC patients improved the overall prognosis (+2 months) of the cohort. These differences can be even more pronounced in immunotherapies or other biological treatments that are more effective in patients with low tumor burden (due to the presence of more immunosuppressing cells in larger masses) [124]. If oligo-metastatic or low-burden disease is not identified, it can impact the effectiveness of the assessment of several drugs in clinical trials. 

We propose that evaluating tumor burden in future clinical trials, including the assessment of OMD and complete tumor volume, would improve the validity of phase III study results, especially in those using biological/immunotherapy drugs. In addition to the standard clinical and radiological evaluations, advanced computational tools and artificial intelligence for automated tumor volume quantification could be integrated into clinical trial designs for better patient stratification and efficacy data interpretation. Furthermore, a harmonized definition and reporting of oligo-metastatic and low-burden diseases through consensus meetings is essential.

## 12. Identification of OMD

Some practical considerations and take-home messages can be made. To date, true de novo OMD definition remains elusive. In fact, a single imaging snapshot of a patient with only a few visible lesions may not be representative of the true tumor burden and evolution. However, from a clinical standpoint, in synchronous OMD (when the primary tumor and sites of metastases are diagnosed simultaneously or <6 months after the treatment of the primary tumor), it is reasonable to suspect a polymetastatic disease. In metachronous OMD, we can hypothesize a more favorable prognosis than synchronous disease because of the lower capacity to develop a metastatic progeny. In the case of induced OMD (patients with a previous history of polymetastases), it is even more difficult to predict the course of the disease. Once again, the definition of OMD mainly relies on a clinical retrospective approach. Oligopersistent and oligoprogressive diseases are very heterogeneous clinical entities with unpredictable clinical behavior requiring an extremely personalized and multidisciplinary diagnostic and therapeutic approach. In any case, the identification of specific OMD biomarkers is a crucial challenge.

## 13. Conclusions

To date, we are aware that not only an elusive “spectrum” of malignancy, but also an accompanying evolving range of potentially curative treatment exist. The interactions between these two concepts will lead, in the near future, to increasingly individualized and integrated cancer treatments with the support of improved molecular assessments and computational tools, including artificial intelligence.

## Figures and Tables

**Table 1:** Incidence of oligo-metastatic patients at diagnosis among metastatic patients in the ten most common cancers.

Cancer	Incidence of Oligo-Metastatic Disease (% on Metastatic Presentation)
Lung (NSCLC)	5
Lung (SCLC)	Undefined (extremely rare)
Breast	5–20
Colorectal	10–15
Stomach	5
HCC	10–40
Prostate	10–30
Pancreatic	5
Ovarian	5–15
Esophageal	5
Cervical	5–15

**Table 2:** Randomized trials including SRT treatment in OMD.

Type of Cancer	Targetable Mutations	Maximum No. of Lesions	Arms	No. of Patients	mPFS (Months)	*p*	mOS (Months)	*p*
NSCLC	Not permitted	6	SRT + CT	14	9.7		NR	
			CT	15	3.5	0.01	17.0	NR
CRC	Permitted	10 liver met	RFA + resection + CT	51	16.8		45.6	
			CT	57	9.9	0.005	40.5	0.01
NSCLC	Permitted	3	SRT or surgery	25	14.2		41.2	
			Observation or CT	24	4.4	0.022	17.0	0.034
Breast, CRC, NSCLC, prostate, others	Permitted	5	SRT + SC	66	12.9		41.0	
			SC	33	6.0	0.0012	28.0	0.09
NSCLC	Only EGFR-mutated cancers	5	SRT + TKI	68	20.2		25.5	
			TKI	65	12.5	<0.001	17.4	<0.001

CRC: colorectal cancer; CT: chemotherapy; met: metastases; mOS: median overall survival; mPFS: median progression-free survival; NSCLC: non-small cell lung cancer; RFA: radio-frequency ablation; SC: Standard of Care; SRT: stereotactic radiotherapy; TKI: tyrosine kinase inhibitor.

## Data Availability

Not applicable.
